# HIV Infection, Risk Factors, and Preventive Services Utilization among Female Sex Workers in the Mekong Delta Region of Vietnam

**DOI:** 10.1371/journal.pone.0086267

**Published:** 2014-01-24

**Authors:** Bach Xuan Tran, Thuong Vu Nguyen, Quang Duy Pham, Phuc Duy Nguyen, Nghia Van Khuu, Nhung Phuong Nguyen, Duc Hoang Bui, Huong Thu Thi Phan, Long Thanh Nguyen

**Affiliations:** 1 Hanoi Medical University, Hanoi, Vietnam; 2 Pasteur Institute, Ho Chi Minh City, Vietnam; 3 University of Texas Health Science Center at Houston, Houston, Texas, United States of America; 4 Authority of HIV/AIDS Control, Ministry of Health, Hanoi, Vietnam; UCL Institute of Child Health, University College London, United Kingdom

## Abstract

**Background:**

Risk behaviors among female sex workers (FSW) are considerable drivers of HIV infections in Vietnam, especially transmission between high-risk and low-risk groups. We assessed HIV prevalence and its correlates among FSWs, and the use of preventive services among this community in the Mekong Delta region, southern Vietnam.

**Methods:**

A cross-sectional survey of 1,999 FSWs was carried out in five provinces including Ben Tre, Hau Giang, Kien Giang, Tien Giang, and Vinh Long between June, 2006 and June, 2007. We interviewed participants face-to-face in order to elicit information about their lives and potential risk factors, and we tested their sera to determine their HIV status. We then performed multivariate logistic regression analyses to investigate factors associated with HIV infection.

**Results:**

Seventeen percent of the participating FSWs were street-based sex workers (SSWs) and the rest (83%) were entertainment establishment-based sex workers (ESWs). Unprotected sex with regular and casual clients in the past month was frequent among study participants (40.5% and 33.5% respectively). However, few respondents (1.3%) had ever injected drugs. Only 2.1% (95% confidence interval (CI): 1.6%–2.8%) of FSWs were found to be infected with HIV. HIV prevalence among SSWs was greater than among ESWs (3.8% vs. 1.8%, p = 0.02, respectively). Increased risk for HIV infection was significantly associated with the number of clients per month (adjusted odd ratio (aOR) = 2.65, 95% CI: 1.26–5.59).

**Conclusions:**

Interventions to reduce unsafe sex and drug injection, and to increase uptake of HIV testing among FSWs are necessary. Differences in HIV prevalence and its correlates by type of sex work emphasize the importance of constrained contexts in shaping risk behaviors among FSWs; that should be considered in designing HIV prevention programs.

## Introduction

Global efforts in the fight against HIV epidemics have resulted in a 25% reduction in HIV incidence [1]. However, in many parts of the world, the burden of HIV remains high among high-risk populations, including female sex workers (FSWs), injecting drug users, and men who have sex with men [2]. It is estimated that approximately one-eighth (11.8%) of FSWs in low and middle-income countries are living with HIV [2]. In addition, a study of FSWs in 20 low and middle-income countries found that only about one-third (34%, range: 4%–79%) of FSWs had ever been tested for HIV, suggesting that comprehensive interventions are necessary to increase access to HIV prevention and care services for this population [3]. However, knowledge of HIV transmission among FSWs in many Asian settings is still limited due to their poor representation in HIV surveillance systems [2].

In Vietnam, prostitution illegal, and many communities view it as immoral or the cause of HIV/AIDS [4]. Thus, women who engage in sex work are highly stigmatized. Among the estimated 300,000 FSWs in the country, HIV prevalence in 2012 was estimated to be 3%, and reached as high as 10% in some provinces [4], [5]. The national HIV sentinel surveillance revealed that unsafe sex and drug injection were fuelling the spread of HIV in this population [6]. Furthermore, sex work places the clients and partners of FSWs at high risk of HIV infection as well, who can then act as a bridge for the disease into the general population [4].

Despite this evidence of HIV transmission among FSWs, there is currently little evidence available about HIV infection among FSWs in the Mekong Delta Region. The Mekong Delta Region is in southwestern Vietnam, which is known for the vast rice fields and plantations that make up the core of its economy. A few studies, with limited numbers of participants, have provided preliminary results about HIV transmission and risk behaviors of FSWs in this region [9]–[11]. For example, heterosexual sex is the dominant mode of HIV transmission, and among FSWs, HIV prevalence was estimated to increase from 5.2% in 2002 to 6.8% in 2011 [8] We were interested in conducting a larger study of FSWs in the Mekong Delta region to assess the prevalence and correlates of HIV infection in this community. In addition, we were interested in the utilization of healthcare services among these women. The public health system in Vietnam is organized into three levels: central, provincial, and grassroots [7]. HIV services are provided by both the public and private sector through both hospitals and preventive care facilities. In addition, there are standalone HIV services delivered by donors and their stakeholders. We conducted a large survey in five provinces to determine HIV prevalence and its correlates, and evaluate access and utilization of HIV preventive and curative services among FSWs in the Mekong Delta Region of Vietnam.

## Methods

### Study design and participant recruitment

A cross-sectional study was conducted in five provinces, Ben Tre, Hau Giang, and Kien Giang, Tien Giang, and Vinh Long, from 06/2007 to 06/2008. Hot-spots of sex work in these provinces were mapped prior to collecting data. We first interviewed key informants, including outreach program officers and peer-educators. Secondary data reviews and field observation were conducted to collect information about names and locations of each hot-spot, and to estimate the size of the target groups. Using peer-educator and outreach staff referrals in order to prevent duplicates, we recruited about 400 FSWs in each province. A total of 1999 FSWs were interviewed, including both current street-based sex workers (SSWs) and entertainment establishment sex workers (ESWs). ESWs include sex workers in restaurants, karaoke, bars/clubs, massage parlors, and SSWs include those who do not work in formal entertainment establishments, but on the street, in alleys, or similar places.

### Measures

FSWs were approached and invited to participate in the study. We interviewed the participants and had their blood samples collected by female health care workers who were experienced in harm reduction programs. The interviews were conducted in private places that were convenient for participants, such as a hotel room or a nearby commune health station. The questionnaire included demographic characteristics, history of sex work, HIV/STI knowledge, attitude and beliefs, self-efficacy, norms, sexual practices and condom use. Blood samples were tested for HIV following the national guideline adopted from the World Health Organization's strategy III for HIV diagnosis.

### Statistical analysis

*In descriptive statistical analysis*, Student-t or Mann-Whitney U tests for continuous variables and Chi-square Fisher exact tests for categorical variables were used to examine the differences across stratified groups when appropriate. Exact binominal method was used to estimate 95% confidence intervals (CI) of HIV infection. *Multivariable logistic regression* was used to assess the association between HIV infection and characteristics of participants. The full model included the following independent variables: demographic characteristics (age, education, marital status), sex work (type of sex work, duration of sex work, selling sex in other provinces or foreign countries, number of clients/month) and known risk factors (drug use, having injecting sexual partners, inconsistent condom use with clients, lovers, and husbands). Using stepwise forward model selection, variables were included when log-likelihood ratio tests gave p-value<0.1, and were excluded at p-values>0.3. Data were managed using Epi Info, and analyzed using Stata version 12.

### Ethical considerations

This research project was led by the Vietnam Authority of HIV/AIDS Control and Pasteur Institute Ho Chi Minh City. Ethical approval was granted by the Ministry of Health, Vietnam. We strictly adhered to the Declaration of Helsinki on ethical principles for medical research. Verbal informed consent was obtained from all participants after a clear explanation of the survey. Participants were able to refuse to participate in and to withdraw from the interview at any time. They were also referred to health care services if necessary. Confidentiality was assured by coding patient information and by securely storing both the paper questionnaires and electronic dataset.

## Results

### Characteristics of study participants

The demographic and sex work characteristics of study participants are presented in Table 1. In brief, participants' age averaged 26.5 years, 47.4% of study individuals completed only elementary school, and 46.8% were single. The majority (83.4%) of the participating FSWs reported alcohol consumption, 3.5% had ever used drugs, and 1.3% had ever injected drugs.

**Table 1:** Demographic and sex work characteristics among study participants.

Characteristics	Vĩnh Long	B 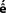 n Tre	Ti 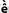 n Giang	H 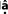 u Giang	Kiên Giang	Total	p-value
**N**	400	400	400	399	399	1,998	
**Demographic characteristics**							
	**Mean (sd)**	**Mean (sd)**	**Mean (sd)**	**Mean (sd)**	**Mean (sd)**	**Mean (sd)**	
**Age (years)**	23.9 (4.7)	30.0 (6.5)	28.2 (8.4)	25.9 (6.1)	24.6 (5.5)	26.5 (6.8)	<0.01
	**N (%)**	**N (%)**	**N (%)**	**N (%)**	**N (%)**	**N (%)**	
**Education**							
Elementary school	153 (38.3)	177 (44.4)	203 (50.8)	206 (51.5)	208 (52.1)	947 (47.4)	<0.01
Secondary school	247 (61.8)	222 (55.6)	197 (49.3)	194 (48.5)	191 (47.9)	1,051 (52.6)	
**Resident status**							
Living alone	110 (27.5)	93 (23.3)	73 (18.3)	64 (16.2)	107 (26.8)	447 (22.4)	<0.01
Living with family/friend(s)	281 (70.3)	300 (75.0)	325 (81.5)	326 (82.3)	291 (72.9)	1,523 (76.4)	
Unstable	9 (2.3)	7 (1.8)	1 (0.3)	6 (1.5)	1 (0.3)	24 (1.2)	
**Marital status**							
Single	275 (68.8)	88 (22.0)	132 (33.0)	206 (51.5)	234 (58.7)	935 (46.8)	<0.01
Married/Living together without marriage	47 (11.8)	101 (25.3)	66 (16.5)	45 (11.3)	25 (6.3)	284 (14.2)	
Divorced/Separated/Widowed	78 (19.5)	211 (52.8)	202 (50.5)	149 (37.3)	140 (35.1)	780 (39.0)	
**Alcohol use**	297 (74.4)	326 (81.5)	298 (74.5)	384 (96.2)	358 (90.2)	1,663 (83.4)	<0.01
**Drug use**	32 (8.1)	15 (3.8)	4 (1.0)	14 (3.5)	5 (1.3)	70 (3.5)	<0.01
**Injecting drug users**	18 (4.5)	1 (0.3)	2 (0.5)	4 (1.0)	1 (0.3)	26 (1.3)	<0.01
**Sex work characteristics**							
	**Mean (sd)**	**Mean (sd)**	**Mean (sd)**	**Mean (sd)**	**Mean (sd)**	**Mean (sd)**	
**Length of selling sex (years)**	2.9 (2.8)	3.2 (3.4)	3.2 (4.1)	2.3 (2.2)	1.9 (2.3)	2.7 (3.1)	<0.01
**Number of partners in the last one month**	18.6 (13.7)	11.9 (6.5)	11.6 (8.5)	15 (11.5)	11 (7.6)	13.6 (10.3)	<0.01
	**N (%)**	**N (%)**	**N (%)**	**N (%)**	**N (%)**	**N (%)**	
**Type of sex work**							
Street based sex workers	58 (14.5)	158 (39.5)	67 (16.9)	37 (9.3)	19 (4.8)	339 (17)	<0.01
Entertainment established sex workers	342 (85.5)	242 (60.5)	330 (83.1)	363 (90.8)	380 (95.2)	1,657 (83)	
**Selling sex in other places**							
In other province(s)	125 (31.3)	95 (23.8)	64 (16.1)	112 (28.1)	93 (23.3)	489 (24.5)	<0.01
Overseas	6 (4.8)	1 (1.1)	2 (3.3)	8 (7.2)	4 (4.3)	21 (4.3)	0.30
**Inconsistent condom use**							
With irregular clients	102 (31.2)	138 (37.4)	54 (18.8)	123 (34.4)	159 (42.3)	576 (33.5)	<0.01
With regular clients	179 (46.0)	202 (53.3)	60 (17.8)	152 (40.3)	134 (42.8)	727 (40.5)	<0.01
With husband/lover	212 (75.4)	206 (78.3)	111 (47.0)	113 (79.0)	128 (77.6)	770 (70.8)	<0.01

Of 1,999 FSWs, 339 (17%) were SSWs and 1,657 (83%) were ESWs. The mean length of sex work was 2.7 years and varied across the five provinces. Participants had on average 13.6 partners in the last month. Approximately one-quarter (24.5%) of the participants reported ever selling sex in places other than the provinces in this study, and a few had sold sex overseas (3.2%). Inconsistent condom use in the past month was the highest with husbands or lovers (70.8%), moderate with regular clients (40.5%) and the lowest with irregular clients (33.5%).

### Prevalence of HIV and health care utilization among participants

The prevalence of HIV among FSWs in Mekong Delta was low (2.1%), and higher among SSWs than among ESWs (3.8% vs. 1.8%, p = 0.02) (Table 2). Among those women who had experienced STI episodes, 56.2% visited public health care services for treatment, and 36.5% used private services; 53.0% reported self-medication, and 53.7% reported abstinence from sexual intercourse. Regarding utilization of HIV services, only 32.7% had ever tested for HIV, and only 54% of these volunteered for testing. ESWs were more likely to have ever been tested (34.4%), however, they were less likely to have done so voluntarily (52.4%). Almost all participants (99.4%) had received at least one HIV prevention service, including condoms (64%) and needle exchange programs (2%). Compared to ESWs, SSWs received more support from peer-educators (62.0%) and civil organization officers (20.4%). Approximately 36.2% of FSWs were referred to STI clinics, and more SSWs (43.8%) were referred than ESWs (34.6%) (Table 2).

**Table 2:** HIV prevalence and healthcare utilizations for HIVby type of sex work.

Characteristics	SSWs (n = 339)		ESWs (n = 1657)		Total		p-value
	n	%	n	%	n	%	
**HIV/STIs infection**							
STI infection in the last 12 months	48	14.2	155	9.4	203	10.2	0.03
HIV infection	13	3.8	29	1.8	42	2.1	0.02
**Healthcare utilization for STIs**							
Do nothing	4	8.3	13	8.4	17	8.4	1.00
Go to public health services	23	47.9	91	58.7	114	56.2	0.19
Go to private health services	22	45.8	52	33.6	74	36.5	0.12
Self-medication through private pharmacy	28	58.3	79	51	107	52.7	0.37
Go to traditional healer	1	2.1	4	2.6	5	2.5	1.00
Self-medication	8	16.7	28	18.1	36	17.7	0.83
Sex abstaining	33	68.8	76	49	109	53.7	0.02
Using condoms	28	58.3	103	66.5	131	64.5	0.30
**Informing partners**	15	31.3	35	22.6	50	24.6	0.22
**Healthcare utilization for HIV**							
**Ever HIV tested**	82	24.4	559	34.4	641	32.7	<0.01
Volunteering for testing	53	64.6	292	52.4	345	54.0	0.11
Knowing the test result	69	84.2	488	87.3	557	86.9	0.43
**Received HIV prevention services**	334	99.7	1,614	99.3	1,948	99.4	0.42
**Types of services**							
Condoms	223	66.8	1,020	63.2	1,243	63.8	0.22
Needles	4	1.2	34	2.1	38	2.0	0.38
Leaflets	201	60.2	928	57.5	1,129	58	0.37
Advices from peer group members	203	60.8	835	51.7	1,038	53.3	<0.01
Advices from peer-educator	207	62	829	51.4	1,036	53.2	<0.01
Advices from health care officers	106	31.7	584	36.2	690	35.4	0.12
Advices from civil organization officers	68	20.4	187	11.6	255	13.1	<0.01
Clubs	20	6.0	76	4.7	96	4.9	0.33
Referrals to STI clinics	146	43.8	559	34.6	705	36.2	<0.01

### Correlates of HIV infections among participants

Bivariate and multivariate regression analyses examined the correlates of HIV infection among FSWs (Table 3). The likelihood of acquiring HIV infection increased significantly in participants with more than 16 clients in the previous month (adjusted Odd Ratio (aOR) = 2.65, p = 0.01), and in participants who exhibited inconsistent condom use with clients in the past month (aOR = 2.08, p = 0.06). Participants who were drug-users or had a drug-using partner were at higher risk of HIV infection than others in the bivariate analysis. Compared to SSWs, ESWs were less likely to be infected with HIV (aOR = 0.46, p = 0.06).

**Table 3:** Correlates with HIV infection among female sex workers.

Independent variables	Bivariate analysis		Multivariate analysis	
	Crude OR (95% CI)	p-value	Adjusted OR (95% CI)	p-value
**Demographic characteristics**				
Age (years)	1.00 (0.96–1.05)	0.83	0.96 (0.89–1.03)	0.28
Education level (Secondary vs. Elementary)	0.74 (0.40–1.37)	0.34		
Marital status (Ref = Single)				
Living with husband/partner	2.59 (1.12–5.97)**	0.03	2.34 (0.72–7.60)	0.16
Divorced/Separated/Widowed	1.77 (0.87–3.61)	0.12	2.44 (0.93–6.40)*	0.07
**Sex work characteristics**				
Type of sex work (RSW vs. SSW)	0.45 (0.23–0.87)**	0.02	0.46 (0.21–1.04)*	0.06
Duration of selling sex (years)	1.02 (0.94–1.12)	0.60		
Working at other place/province (Yes vs. No)	1.24 (0.63–2.44)	0.53		
Number of clients in the last month (>16 clients vs. <16 clients)	2.97 (1.61–5.51)***	<0.01	2.65 (1.26–5.59)**	0.01
**Substance use**				
Injecting drug user (Yes vs. No)	6.47 (1.86–22.45)***	<0.01		
Alcohol use (Yes vs. No)	1.49 (0.58–3.82)	0.41		
Had injecting drug clients (Yes vs. No)	0.94 (0.20–4.41)	0.94		
Had injecting drug husband/lover (Yes vs. No)	6.32 (2.03–19.73)***	<0.01		
**Inconsistent condom use**				
Inconsistent condom use with clients (Yes vs. No)	2.17 (1.03–4.60)**	0.04	2.08 (0.97–4.44)*	0.06
Inconsistent condom use with husband (Yes vs. No)	1.06 (0.44–2.57)	0.89		
**Access to Information sources**				
Reading paper (Yes vs. No)	0.80 (0.43–1.47)	0.47		
Listening radio (Yes vs. No)	0.86 (0.46–1.60)	0.62		
Watching TV (Yes vs. No)	1.50 (0.46–4.91)	0.50		
IEC attendance (Yes vs. No)	0.76 (0.38–1.52)	0.44		
Constant	0.02 (0.01–0.06)***	<0.01	0.02 (0.00–0.37)***	0.01
Observations	1,998		1,518	

*** p<0.01,

** p<0.05,

* p<0.1.

## Discussion

We found a high prevalence of HIV infection risk-factors among FSWs in the Mekong Delta Region of Vietnam, including inconsistent condom use with clients and low access to HIV prevention services. SSWs were found to have a higher risk of HIV infection than ESWs, and also received more interventions. Although very few of the participants were injection drug users, they had significantly higher risk of HIV infection than the participants who were not drug-users.

Interestingly, despite the high prevalence of HIV infection risk factors, the prevalence of HIV infection among FSWs in this study was lower than that of the national surveillance and other provinces [5], [9], [10], [12], [13]. In metropolitan cities such as Hanoi and Ho Chi Minh, 15% and 24% of FSWs were diagnosed HIV positive, and among those, 32% and 12% FSWs had ever used drugs, respectively [6], [13]. Other findings of this study are in line with previous work, however, including the risk factors that increase the vulnerability to HIV of FSWs, such as inconsistent condom use, illicit drug use, and the number of clients [9], [14]–[19]. Understanding these behavioral risk factors is particularly important given that there is substantial evidence about the interactive relationship between these behavioral factors; for example, drug use increases inconsistent condom use among FSWs [9]. Therefore, HIV transmission among FSWs might be driven and exacerbated by the intersection of unsafe sex and needle sharing [2].

It has also been documented that, in various settings, structural risk factors are limiting efforts to control the spread of HIV among FSWs. These structural risk factors include the organization of sex work, regulatory policies, poverty, discrimination and gender inequality [2]. This study contributed to the research on this subject by demonstrating that SSWs had a higher risk of HIV infections than ESWs, a finding that is comparable those in previous studies [9], [11], [12]. Many SSWs come from lower socioeconomic backgrounds than ESWs, have fewer norms towards practicing safe sex, and a higher level of economic pressure. A study conducted in Ho Chi Minh city reported that SSWs earned less money from sex work than ESWs, thus, to receive additional money from clients, they did not use condoms during sexual intercourse [12]. Furthermore, these structural factors - especially discrimination and gender inequality - indirectly heighten risk of HIV infection among FSWs by limiting their ability access to HIV and STI preventive healthcare and treatment. Given their risk behaviors, the utilization of HIV preventive programs and treatment services among FSWs in Mekong delta region is pretty low, especially for needle program. The low level accessibility to HIV prevention and treatment resources among this group is partly due to the fact that FSWs have been less visible to the policy makers and planners than male IDUs [20]. Furthermore, prostitution and illicit drug use are widely considered to be “social evils,” which result in social stigma towards FSWs [9]. These contextual barriers to access to and utilization of HIV interventions must be addressed in order to improve the outcomes of HIV programs among FSWs.

Implications of this study include the need to scale up harm reduction interventions among FSWs in the region. Outreach programs should be tailored specifically for different types of sex work, and should target both unsafe sex and drug injection. Encouraging condom use, voluntary HIV testing, and early access to health care services are necessary to reduce the risk of HIV transmission among FSWs, especially those who are socioeconomically disadvantaged. Finally, the current 100% condom use program should be maintained, along with communication campaigns targeting FSWs and their clients and partners.

This study has some shortcomings. First, participants may have underreported risk behaviors (e.g. drug use, alcohol use, inconsistent condom use) due to participants' attitudes, motivation, and cultural ideas and norms about disclosing sensitive personal information, including sexual and substance use behaviors [12]. Second, causal inferences about risk factors of HIV infection might not be confirmed given the limitations of a cross-sectional study. Notwithstanding, this is the first large scale survey among FSWs in these five provinces of the Mekong Delta Region in Vietnam. Moreover, the mapping of hot-spots enhanced the representativeness of the findings.

## Conclusion

In conclusion, behavioral and structural risk factors have been driving HIV transmission among FSWs in the Mekong Delta region. Interventions targeting FSWs should focus on changing their risk behaviors - both unsafe sex and drug injection - and encouraging HIV testing and early access to preventive services in the region.
